# Cytokine signatures differentiate systemic sclerosis patients at high versus low risk for pulmonary arterial hypertension

**DOI:** 10.1186/s13075-022-02734-9

**Published:** 2022-02-09

**Authors:** Kathleen D. Kolstad, Avani Khatri, Michele Donato, Sarah E. Chang, Shufeng Li, Virginia D. Steen, Paul J. Utz, Purvesh Khatri, Lorinda Chung

**Affiliations:** 1 Department of Medicine, Division of Immunology and Rheumatology, Stanford University School of Medicine, Palo Alto, CA USA; 2 Department of Medicine, Division of Rheumatology, University of California Los Angeles, Los Angeles, CA USA; 3 Institute for Immunity, Transplantation, and Infection, Stanford University, Palo Alto, CA USA; 4 Department of Dermatology, Stanford University School of Medicine, Palo Alto, CA USA; 5 Division of Rheumatology, Georgetown University Medical Center, Washington, DC USA; 6 Department of Medicine, Center for Biomedical Informatics Research, Stanford University, Palo Alto, CA USA; 7 Department of Medicine, Division of Rheumatology, Palo Alto VA Health Care System, Palo Alto, CA USA

**Keywords:** Systemic sclerosis, Pulmonary arterial hypertension, Biomarkers

## Abstract

**Background:**

Pulmonary arterial hypertension (PAH) affects approximately 10% of patients with systemic sclerosis (SSc) and is a leading cause of death. We sought to identify serum cytokine signatures that risk stratify SSc patients for this potentially fatal complication.

**Methods:**

Subjects at high risk for PAH and with incident PAH based on right heart catheterization (RHC) were enrolled in the multi-center prospective registry, Pulmonary Hypertension Assessment and Recognition of Outcomes in Scleroderma (PHAROS). Low-risk SSc patients were enrolled at Stanford and had normal pulmonary function test and echocardiogram parameters. Serum was available from 71 high-risk patients, 81 incident PAH patients, 10 low-risk patients, and 20 healthy controls (HC). Custom 14- and 65-plex arrays were used for cytokine analysis. Cytokine expression was compared between patient groups by principal component analysis and Tukey’s test result. A multiple hypotheses corrected *p* value <0.05 was considered significant.

**Results:**

Exploratory analysis using principal components showed unique clustering for each patient group. There was a significant difference in cytokine expression in at least one group comparison for every cytokine. Overall, there was very little difference in cytokine expression comparing high-risk and PAH patient groups; however, these groups had substantially different cytokine profiles compared to low-risk patients and HC.

**Conclusion:**

These data suggest that cytokine profiles can distinguish SSc patients who are at high-risk for or have PAH from SSc patients who may be at lower risk for PAH and HC. However, high-risk and PAH patients had very similar cytokine profiles, suggesting that these patients are on a disease continuum.

**Supplementary Information:**

The online version contains supplementary material available at 10.1186/s13075-022-02734-9.

## Background

Systemic sclerosis (SSc) is an autoimmune rheumatic disease characterized by vasculopathy, immune system dysregulation, and fibrosis of the skin and internal organs. Pulmonary arterial hypertension (PAH) is a leading cause of death in patients with systemic sclerosis, affecting 8-12% of this patient population [1, 2]. There is accumulating evidence that early PAH-specific therapy can improve survival and functional status in patients with SSc [3]. Early PAH screening and diagnosis has also been suggested to improve outcomes [4, 5]. Right heart cathetherization (RHC) is the gold standard for the diagnosis of PAH. Given the invasive nature of this procedure, a variety of screening algorithms have been implemented to identify SSc patients at high risk for PAH and who warrant early referral to RHC. However, although many of the commonly used algorithms have high sensitivity (97–100%), they suffer from low specificity (26–55%) and marginal positive predictive value (60–67%) [6].

There has been increasing interest in identifying cytokines as biomarkers for disease severity and progression in patients with SSc and SSc-PAH. Furthermore, recognizing dysregulated cytokines has the potential to help understand the pathogenesis of disease and could identify therapeutic targets. Previous studies have detected changes in expression of individual cytokines involved in vascular injury and inflammation in patients with SSc-PAH compared to healthy controls and patients with SSc and no PAH. More specifically increases in inflammatory mediators such as TNF-alpha, IL1-beta, ICAM-1, and IL-6, and markers of vascular injury such as VCAM-1, VEGF, and von Willebrand factor have been identified in patients with SSc-PAH [7].

In the current study, we aimed to characterize specific cytokine signatures that differentiate patients with incident SSc-PAH, patients at high risk for SSc-PAH, patients at low risk for SSc-PAH, and healthy controls. We anticipate these data will assist with the early identification of patients at high risk for or with incident SSc-PAH. Additionally, we expect this study will identify cytokines that may be involved in the pathogenesis of SSc-PAH and could serve as therapeutic targets.

## Materials and methods

### Patient population

Clinical data and serum samples for PAH and high-risk PAH patients are from the Pulmonary Hypertension Assessment and Recognition of Outcomes in Scleroderma (PHAROS) study, a US-based, multi-center registry of SSc patients at high risk for (high-risk PAH) or with incident PAH confirmed by RHC within 6 months of enrollment. Clinical data and serum samples were collected from 2006 to 2016. For this study, the Institutional Review Board at 22 participating US centers approved the PHAROS protocol and patients provided written informed consent prior to enrollment.

Incident PAH patients had a RHC within 6 months of enrollment demonstrating mean pulmonary artery pressure (mPAP) ≥25 mmHg, pulmonary capillary wedge pressure (PCWP) ≤15 mmHg, and no significant interstitial lung disease as determined by chest imaging and/or a forced vital capacity (FVC) >60% predicted. High-risk PAH patients were considered to be at risk for development of definitive PAH and were defined as having any one of the following features: (1) diffusing capacity of carbon monoxide (DLCO) <55% predicted with a FVC of >70% predicted, (2) FVC/DLCO ratio >1.6, or (3) estimated right ventricular systolic pressure (RVSP) ≥40 mmHg on echocardiography at the time of enrollment. There were 81 incident PAH patients and 71 high-risk PAH patients included in the current study.

Patients with SSc considered to be low-risk for SSc-PAH had a DLCO ≥ 80%, FVC ≥ 80%, and RVSP ≤35mmHg or normal echocardiogram if no measurable tricuspid regurgitant jet was observed at the time of enrollment (*n*=10). This patient group was enrolled at the Stanford Rheumatology Clinic. Healthy controls (HC) (*n*=20) were enrolled through the Stanford rheumatology-dermatology clinic and they had no known autoimmune disease.

Baseline demographics and clinical characteristics were compared between the four patient groups, including age, gender, race, cutaneous subtype (limited versus diffuse), autoantibody subtype, disease duration, pulmonary function test findings (FVC% predicted, DLCO % predicted), and RVSP on echocardiogram. All serum samples were drawn at the time of disease risk classification, at patient enrollment or in the case of incident SSc-PAH patients, within 6 months of diagnostic RHC.

### Cytokine expression profiling and analysis

Serum samples were obtained from the above-described patient groups. Sample collection and storage was standardized across study sites. Blood samples were collected in red top tubes and centrifuged at the end of the clotting time (30–60 min) for 20 min at 1100–13,000*g* at room temperature. Serum was stored in aliquots at 80 °C. Cytokines chosen for analysis were based on cytokines assessed in early feasibility studies using core facility commercial arrays (Supplementary Table 1). The cytokines assessed in these early studies were limited by assay availability at the facility. A custom 14-plex array and the Immune Monitoring 65-Plex Human ProcartaPlex™ Panel array from Invitrogen were used for cytokine analysis to include all cytokines of interest. Samples were diluted 1:100 in assay buffer and run in duplicate. Diluted samples were incubated with magnetic beads for 1 hour. Beads were washed twice and incubated with detection antibody for 30 min, followed by two washes and incubation with Streptavidin-PE for 30 mins. Beads were washed twice, resuspended in reading buffer, and analyzed on the Luminex Flexmap 3D system. Samples were normalized by background correction and removed if the correlation between technical replicates was <0.8. Mean values and standard deviations for each cytokine by the patient group can be found in Supplementary Table 2. Cytokine expression was compared between patient groups using Tukey’s test. A multiple hypotheses corrected *p* value <0.05 was considered significant. The complex heatmap shows the magnitude of the fold changes between conditions and their statistical significance for each comparison. Significance was based on the false discovery rate (FDR) threshold of 5%.

## Results

### Baseline characteristics patient population

Baseline characteristics of each patient group are described in Table 1. The majority of patients in all groups were women. The majority of patients in the PAH and high-risk PAH groups, but only a third of patients in the low-risk group, were Caucasian. Fifty percent of low-risk SSc group had limited disease and 76% and 71% of high-risk and PAH patients had limited disease, respectively. Anti-centromere (25%) and isolated nucleolar (25%) autoantibody subtypes were most common in the PAH group, while anti-centromere (21%) and Scl-70 (21%) were most common in the high-risk PAH group, and anti-centromere (60%) was most common in the low-risk group. Disease duration was similar among all SSc groups. After 3 years of follow-up, no patient in the low-risk SSc patient group had been diagnosed with PAH. The median and range % predicted FVC, % predicted DLCO, and RVSP values at baseline for the low-risk SSc group were 96 (84–112), 99 (88–124), and 28mmHg (18–35 mmHg), respectively. At follow up the median and range % predicted FVC, % predicted DLCO, and RVSP values were 90 (74–112), 95 (59–116), and 30 mmHg (20–39mmHg), respectively. The patient who developed an RVSP >35 mmHg passed away from metastatic cancer 3 months after an echocardiogram was performed.

**Table 1:** Baseline characteristics of patient groups

	**Healthy**	**PAH**	**High risk**	**Low risk**	***p*value**
	***n*=20**	***n*=81**	***n*=71**	***n*=10**	
**Age, mean (std) (*n*)**	59.7 (8.9) (20)	58 (11.3) (79)	60.1 (10.8) (71)	52.3 (10.7) (10)	0.20
**Sex**					0.18
Female	15 (75.0)	59 (74.7)	62 (87.3)	9 (90.0)	
Male	5 (25.0)	20 (25.3)	9 (12.7)	1 (10.0)	
**Race/ethnicity**					<.0001
Asian/Pacific Islander		1 (1.3)	1 (1.4)	3 (30.0)	
Black		12 (15.2)	2 (2.8)	0	
Caucasian		64 (81.0)	60 (84.5)	3 (30.0)	
Hispanic		2 (2.5)	5 (7.0)	3 (30.0)	
Native American		0	2 (2.8)	0	
Other Ethnic Origin		0	1 (1.4)	1 (10.0)	
**SSc subtype**					0.39
Diffuse		20 (25.0)	15 (21.1)	5 (50.0)	
Limited		57 (71.2)	54 (76.1)	5 (50.0)	
Unclassified		3 (3.8)	2 (2.8)	0	
**Antibody**					0.1433
Mixed or other		15 (19.0)	13 (18.6)	0	
Scl 70		13 (16.5)	15 (21.4)	2 (20.0)	
U1RNP		3 (3.8)	1 (1.4)	0	
Centromere		20 (25.3)	15 (21.4)	6 (60.0)	
Isolated nucleolar		20 (25.32)	13 (18.57)	0	
Negative		3 (3.8)	9 (12.9)	2 (20.0)	
RNA polymerase III		5 (6.3)	4 (5.7)	0	
**FVC, median (range) (n)**		71.2 (27.1–104.8) (69.0)	85.2 (32.5–130.6) (64.0)	96.0 (84.0–112.0) (10.0)	<.0001
**DLCO, median (range) (n)**		37.0 (9.9–90.7) (67)	50.4 (10.1–94.2) (60)	98.5 (80.0–128.0) (10)	<.0001
**RVSP, median (range) (n)**		50.5 (17.0–120.0) (68)	38.5 (23.0–80.0) (60)	28.0 (18.0–35.0) (7)	<.0001
**Disease duration, years, median (range) (*n*)**					
Raynaud’s symptom		10.2 (0.9–45.1) (75)	10.7 (0.4–49.2) (64)	7.3 (1.5–33.7) (9)	0.4951
Non-Raynaud’s symptom		8.7 (0.0–33.0) (74)	9.3 (0.1–40.9) (66)	5.1 (1.3–15.3) (9)	0.1644

*SSc* systemic sclerosis, *PAH* pulmonary arterial hypertension, *ANA* antinuclear antibodies, *FVC* forced vital capacity, *DLCO* diffusing capacity for carbon monoxide, *RVSP* right ventricular systolic pressure

### Cytokine Array Results

**Fig. 1:**
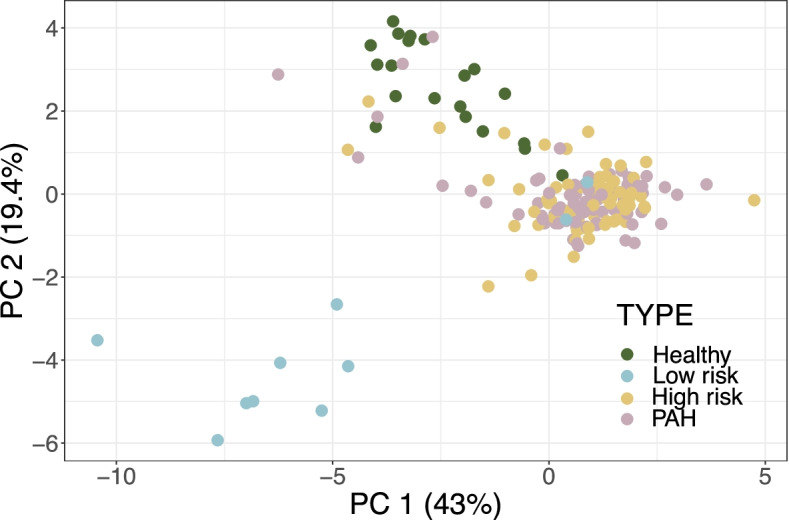
Principal component analysis plot of 14-plex cytokine array data shows all 182 samples along PC1 and PC2, which represent 43% and 19.4% of the variability, respectively, within the data. PCA plot distinguished different patient groups. Healthy controls and low-risk SSc patients were different from SSc patients with PAH or at high risk of developing PAH. SSc, systemic sclerosis; PAH, pulmonary arterial hypertension

**Fig. 2:**
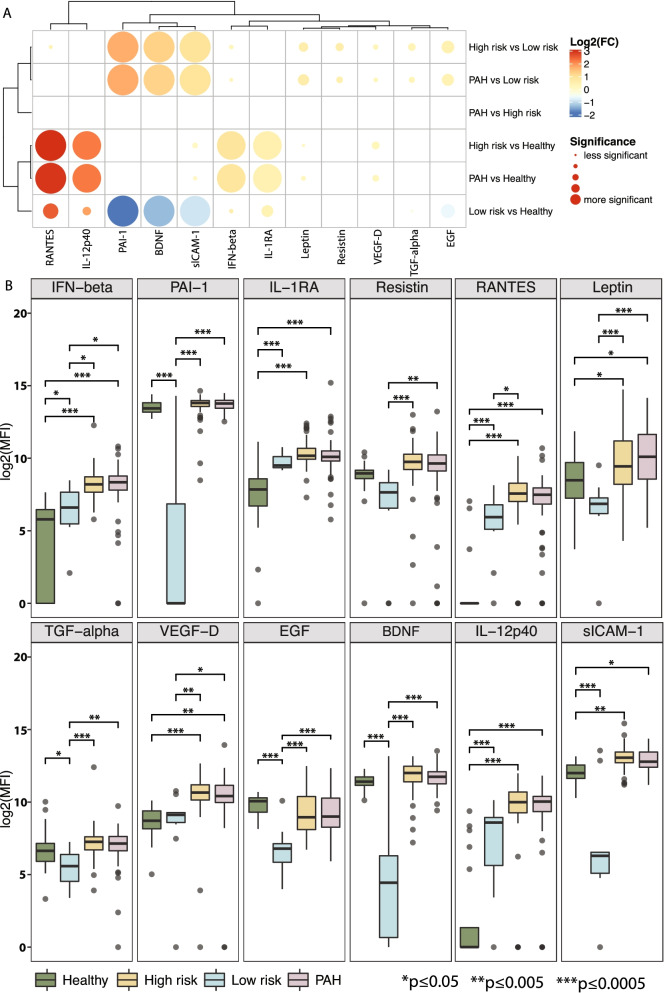
**A** Multiple hypotheses corrected p-values for each antigen in every pairwise comparison using Tukey’s test. This heatmap shows the magnitude of the fold changes between conditions and their statistical significance for each comparison. The color of each circle represents the fold change between conditions, where red indicates a high fold change, light yellow a low fold change, and blue indicates negative fold change. The size of each circle is proportional to the statistical significance of the difference between conditions, where larger circles represent more significant differences. A white cell represents antigens that were not showing a statistically significant difference between conditions based on the FDR threshold of 5%. **B** Boxplots of expression of each significant antigen in each of the four groups. Boxes represent inter-quartiles (25% and 75% percentile), and whiskers represent maximum and minimum values. SSc, systemic sclerosis; PAH, pulmonary arterial hypertension

Two cytokines (sVCAM-1 and PDGF-BB) from the 14-plex cytokine array and 22 from the 65-plex cytokine array were removed from further analysis due to low correlation between technical replicates. Principal component analysis (PCA) was used as dimensionality reduction and visualization technique for exploratory analysis of the data. PCA represents an orthogonal transformation of series of potentially coordinated observations into principal components. Typically, the first two to three principal components explain the majority of the variance in data and are used to visualize data in 2- or 3-dimensions. In our analysis, PCA showed unique clustering for each patient group in both arrays. This finding was particularly evident in the 14-plex cytokine array data (Fig. 1A and Supplementary Fig. 1). Data was analyzed using analysis of variance (ANOVA) and Tukey’s honestly significant difference (HSD) test for post hoc comparisons [8]. The heatmaps show the magnitude of the fold changes between conditions and their statistical significance for each comparison. For the 14-plex array data, there was very little difference in cytokine expression comparing high-risk and PAH patient groups; however, these groups had substantially different cytokine profiles compared to low-risk patients and HC patients. In particular, RANTES, IL-12p40, IFN-beta, and IL-1RA were significantly higher in patients with PAH and both high-risk and low-risk patients compared to healthy controls (Fig. 2A, B). Importantly, PAI-1, BDNF, sICAM-1, and EGF were significantly higher in the high-risk and PAH groups compared to the low-risk group, were but significantly lower in the low-risk group compared to healthy controls (Fig. 2A, B). Leptin and VEGF-D were also significantly higher in the high-risk and PAH groups compared with healthy controls and low-risk patients (Fig. 2A, B).

Principal component analysis of the 65-plex array showed the most prominent differences in cytokine profiles when comparing the high-risk and incident PAH patients with HC patients (Supplementary Fig. 1). Similar to the 14-plex array results, the PAH and high-risk PAH patient groups had very similar cytokine expression profiles. Each of the 43 cytokines was significantly higher in patients with PAH or high-risk patients compared to HCs, whereas 27 of those were significantly higher in low-risk patients compared to healthy controls (Supplementary Fig. 2A-B). Importantly, IL3, MCSF, ENA78, Eotaxin3, and TNFRII were significantly higher in patients with PAH and high-risk patients compared with low-risk patients. However, there were also multiple cytokines that distinguished all SSc groups from HC patients. (Supplementary Fig. 2A-B).

## Discussion

The objective of the current study was to identify distinct cytokine profiles that can discriminate SSc patients based on PAH status. Our data suggest that cytokine profiles can differentiate SSc patients who are at high-risk for or have PAH from SSc patients at low risk for PAH and healthy controls. However, high-risk and PAH patients had very similar cytokine profiles, suggesting that these patients are on a disease continuum.

Similar to prior studies, we found elevated levels of inflammatory mediators in SSc patients with PAH and high-risk for PAH such as TNF-alpha and IL-6 compared to healthy controls [7, 9]. There was no difference in TNF-alpha levels when comparing low-risk and healthy patients. However, in the current study, these cytokines did not distinguish PAH and high-risk patients from low-risk patients (Fig. 2A and Supplementary Fig. 2A). Our study extended these findings and noted prominent increases in pro-inflammatory cytokines including RANTES, IL-12p40, and IFN-Beta, in patients with SSc overall, but most prominently in patients on the PAH spectrum when compared to healthy controls. Changes in these cytokines have been associated with pulmonary hypertension previously [10, 11]. Consistent with prior studies, our data also show evidence of vascular dysregulation in the significantly increased levels of VEGF-D in the high-risk and PAH groups compared to low risk and HC groups [7, 8].

A major strength of this study is the comparison of cytokine profiles between an SSc group at low-risk for PAH based on well-established clinical parameters with patients at high risk for or with PAH [2]. Being able to use biomarkers to predict the devastating complication of PAH has the potential to identify patients who may benefit from aggressive screening and early therapy. Although it is unknown whether these low-risk patients may ultimately develop PAH over time, they did not have evidence of disease three years after serum samples were drawn and had similar disease duration compared to SSc patients on the PAH spectrum. Cytokines most prominently differentiating high-risk and PAH patients from low-risk SSc patients were PAI-1, sICAM-1, BDNF, and VEGF-D. PAI-1 and sICAM-1 are cytokines implicated in modulating fibrosis and endothelial cell function and have been shown to have abnormal levels in patients with SSc [12]. BDNF may play an important role in modulating angiogenesis and sympathetic function and there is evidence that hypoxia can increase the levels of BDNF production from pulmonary artery endothelial cells [13]. Consistent with this, we found that BDNF levels were elevated in high-risk and PAH patients compared to low-risk patients. We also found that BDNF levels were lower in low-risk patients compared to healthy controls. This is in line with prior studies that showed BDNF levels in an SSc population enriched with those without PAH was lower than controls [14].

It is notable that PAI-1, BDNF, and sICAM-1 were significantly lower in the low-risk group compared to the healthy controls and significantly elevated in the high-risk and PAH populations compared to low-risk patients. Dysregulation of these cytokines may occur with the development of SSc, perhaps related to hypoxic injury. With the development of pulmonary vascular disease, persistent hypoxia may induce a negative feedback loop resulting in pronounced elevation of these cytokines.

A major strength of this study is that samples and clinical data were obtained from a large prospective multi-center registry of SSc patients enrolled at scleroderma centers that routinely screen their patients for PAH. However, the number of low-risk patients in the comparator group was limited as they were recruited from a single center. The patients with PAH were diagnosed using the gold standard of RHC. However, the high-risk patients underwent RHC only if deemed clinically appropriate by the treating physician. Furthermore, since the closure of this registry, criteria for the diagnosis of PAH have been revised with a lower mPAP (>20mmHg) [15]. As such, many of the high-risk PAH patients included in the study may in fact meet criteria for PAH. Regardless of the mPAP cut-off for the diagnosis of PAH, our findings of similar cytokine profiles observed between the high-risk and incident PAH groups supports a continuum of disease.

Although we assessed many cytokines, there are some cytokines that have distinguished SSc-PAH patients in other studies that were not included based on available data at the time of assay preparation and limitations of the assays themselves [16]. Another limitation of this study is that we are unable to make any conclusions about the unique cytokine milieu of the pulmonary vascular bed that may contribute to PAH given the results are based on serum studies. Finally, these results need to be validated in an independent cohort and with a larger low-risk SSc patient group.

## Conclusions

This study illustrated distinct cytokine profiles that can distinguish SSc patients who are at high-risk for or have PAH from SSc patients who are at low risk for PAH and HC. We anticipate these data could be used to risk stratify SSc patients and guide therapy. These data may provide potential mechanistic targets to increase our understanding of SSc-PAH and support therapeutic innovation in the future.

## Supplementary Information

**Additional file 1: Supplementary Figure 1.** Principal component analysis plot of 65-plex cytokine array data shows all 182 samples along PC1 and PC2, which represent 63.4% and 4.6% of the variability, respectively, within the data PCA plot distinguished different patient groups. Healthy controls and low risk SSc patients were different from SSc patients with PAH or at high risk of developing PAH. SSc=systemic sclerosis; PAH=pulmonary arterial hypertension.

**Additional file 2: Supplementary Figure 2.** 65-plex cytokine array results. (A) Multiple hypotheses corrected p-values for each antigen in every pairwise comparison using Tukey’s test. Every antigen was significantly different in at least one comparison. No antigen was significantly different between SSc patients at high risk of PAH or with PAH. (B) Boxplots of expression of each significant antigen in each of the four groups. Boxes represent inter-quartiles (25% and 75% percentile), and whiskers represent maximum and minimum values Number of * indicate p-value by Tukey’s HSD post hoc: *P≤0.05; **P≤0.005; ***P≤0.0005). SSc=systemic sclerosis; PAH=pulmonary arterial hypertension.

**Additional file 3: Supplementary Table 1.** Cytokine abbreviations.

**Additional file 4: Supplementary Table 2.** Mean/standard deviation values for each cytokine by clinical group.

## Data Availability

The datasets generated during the current study is publicly available from the NCBI. The GEO accessions numbers are GSE195581 and GSE195583.

## Abbreviations

SSc: Systemic sclerosis
PAH: Pulmonary arterial hypertension
PHAROS: Pulmonary hypertension assessment and recognition of outcomes in scleroderma
HC: Healthy controls
RHC: Right heart catheterization
mPAP: Mean pulmonary artery pressure
PCWP: Pulmonary capillary wedge pressure
FVC: Forced vital capacity
DLCO: Diffusing capacity of carbon monoxide
RVSP: Right ventricular systolic pressure
FDR: False discovery rate
