# Supplementary figures and images for: Cytokine signatures differentiate systemic sclerosis patients at high versus low risk for pulmonary arterial hypertension
Source: Arthritis Res Ther. 2022 Feb 9;24:39. doi: 10.1186/s13075-022-02734-9 (PMC8827262; doi:10.1186/s13075-022-02734-9)

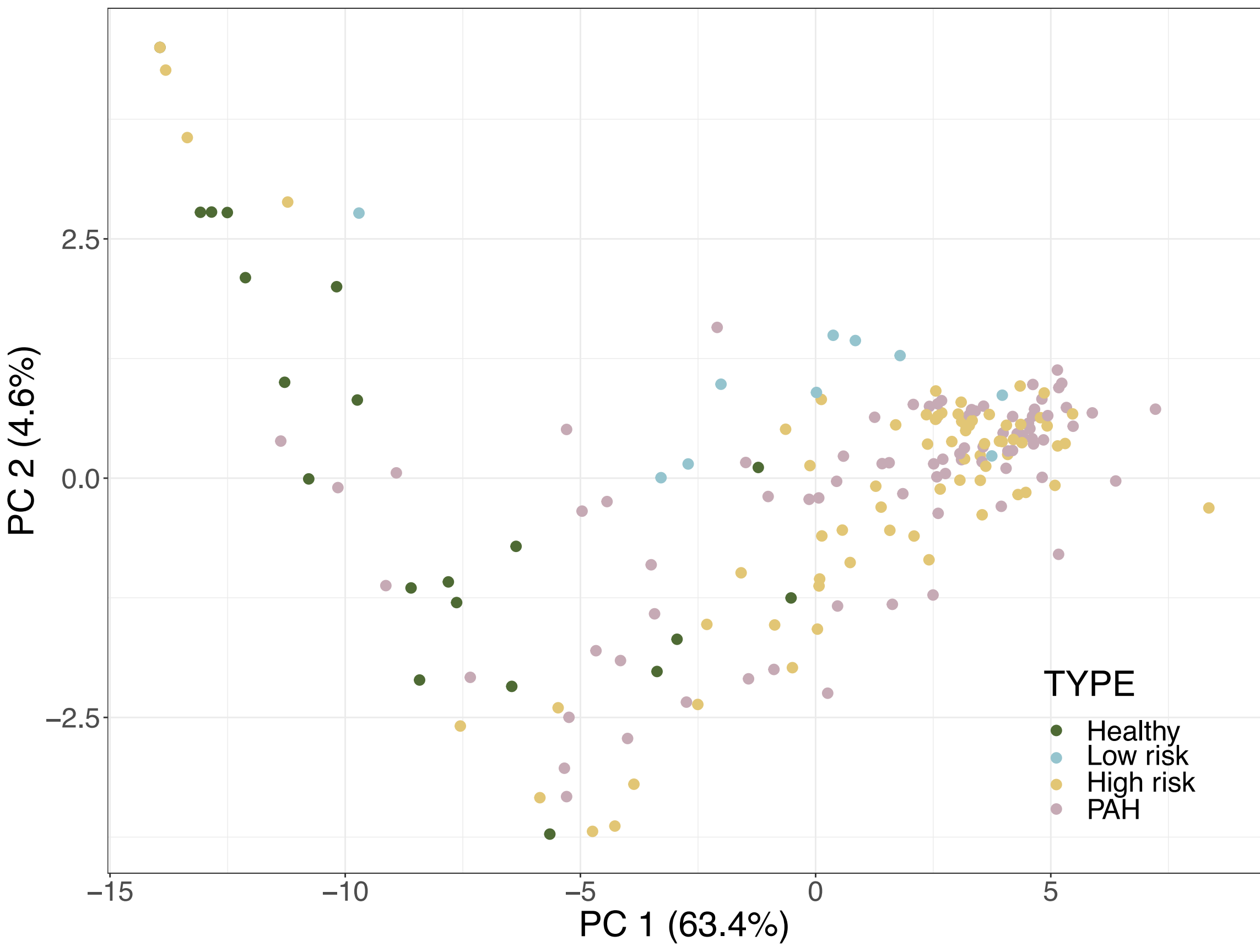

Supplement: Supplementary file 1 — Additional file 1: Supplementary Figure 1. Principal component analysis plot of 65-plex cytokine array data shows all 182 samples along PC1 and PC2, which represent 63.4% and 4.6% of the variability, respectively, within the data PCA plot distinguished different patient groups. Healthy controls and low risk SSc patients were different from SSc patients with PAH or at high risk of developing PAH. SSc=systemic sclerosis; PAH=pulmonary arterial hypertension. [file 13075_2022_2734_MOESM1_ESM.pdf]

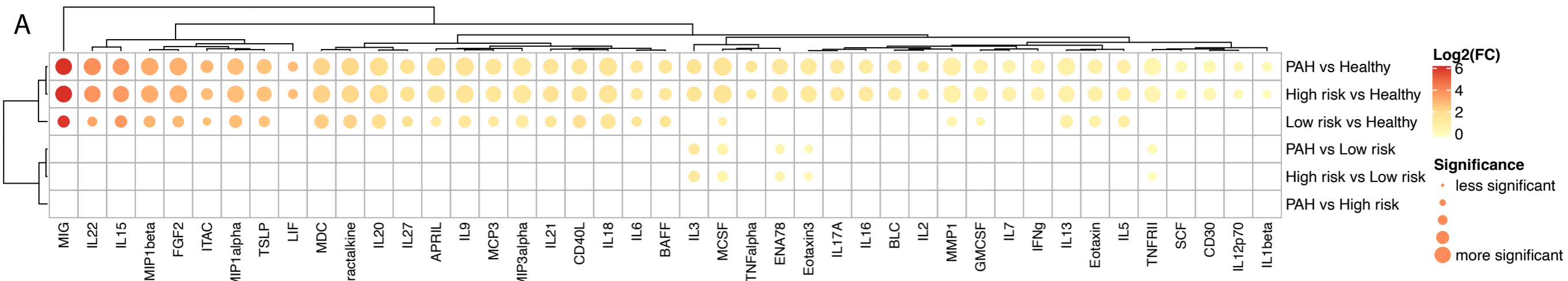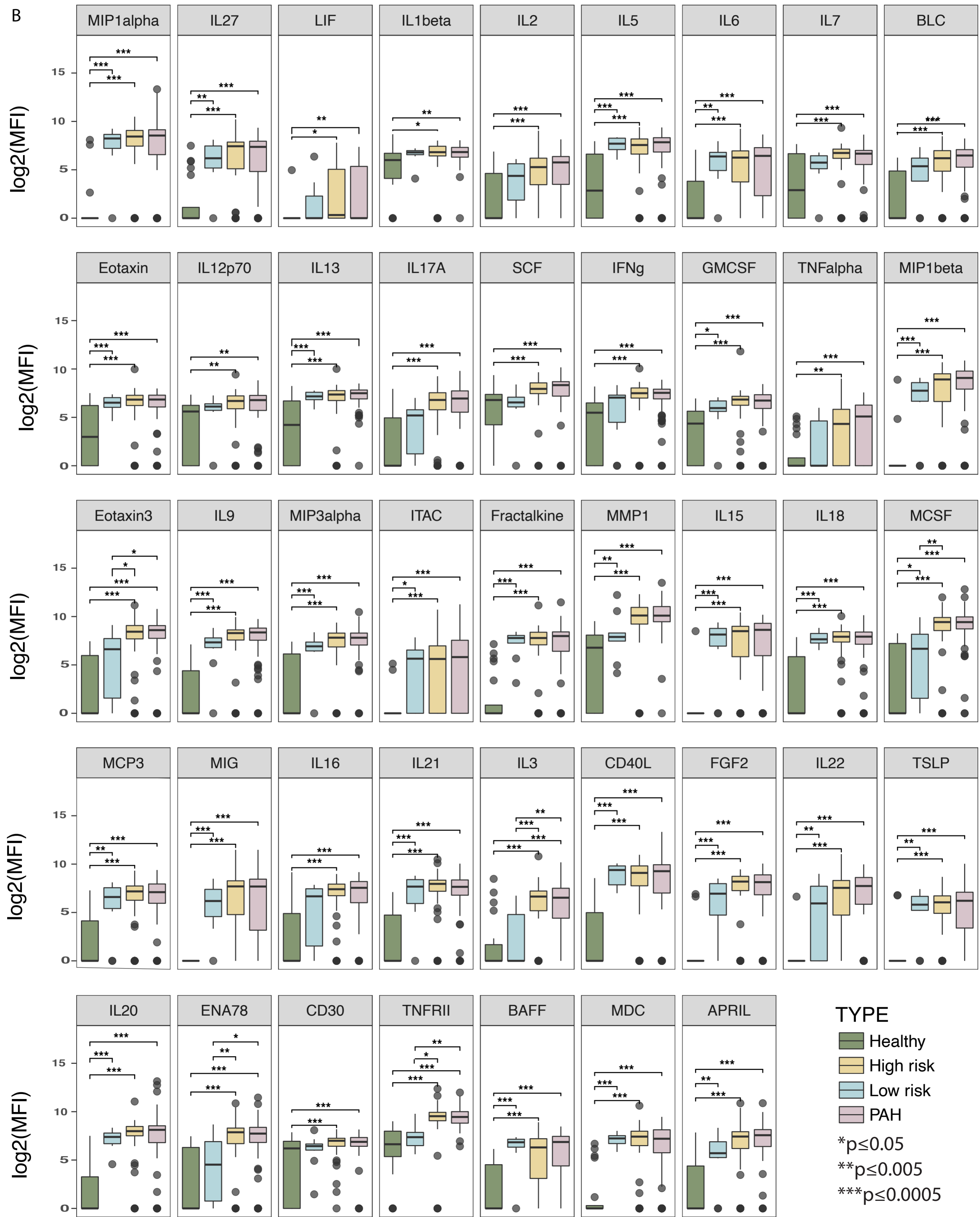

Supplement: Supplementary file 2 — Additional file 2: Supplementary Figure 2. 65-plex cytokine array results. (A) Multiple hypotheses corrected p-values for each antigen in every pairwise comparison using Tukey’s test. Every antigen was significantly different in at least one comparison. No antigen was significantly different between SSc patients at high risk of PAH or with PAH. (B) Boxplots of expression of each significant antigen in each of the four groups. Boxes represent inter-quartiles (25% and 75% percentile), and whiskers represent maximum and minimum values Number of * indicate p-value by Tukey’s HSD post hoc: *P≤0.05; **P≤0.005; ***P≤0.0005). SSc=systemic sclerosis; PAH=pulmonary arterial hypertension. [file 13075_2022_2734_MOESM2_ESM.pdf]
