# Supplementary material for: Cytokine signatures differentiate systemic sclerosis patients at high versus low risk for pulmonary arterial hypertension
Source: Arthritis Res Ther. 2022 Feb 9;24:39. doi: 10.1186/s13075-022-02734-9 (PMC8827262; doi:10.1186/s13075-022-02734-9)
Supplement: Supplementary file 3 — Additional file 3: Supplementary Table 1. Cytokine abbreviations. [file 13075_2022_2734_MOESM3_ESM.docx]

| Cytokine Abbreviation | Cytokine | Cytokine Abbreviation | Cytokine | Cytokine Abbreviation | Cytokine |
| --- | --- | --- | --- | --- | --- |
| BDNF | Brain-derived neurotrophic factor | APRIL | A proliferation-inducing ligand | HGF | Hepatocyte growth factor |
| EGF | Epidermal growth factor | BAFF | B-cell activating factor | IFN-alpha | Interferon-alpha |
| ICAM-1 | Intercellular Adhesion Molecule 1 | BLC | B lymphocyte chemoattractant | IFN-gamma | Interferon-gamma |
| IFN beta | interferon beta | CD30 | CD30 | IL-1 alpha | Interleukin 1 alpha |
| IL-1RA | interleukin-1 receptor antagonist | CD40L | CD40 ligand | IL-1 beta | Interleukin 1 beta |
| IL-12/IL-23p40 | Interleukin-12/Interleukin-23p40 | ENA-78 | C-X-C motif chemokine 5 | IL-10 | Interleukin-10 |
| Leptin | Leptin | Eotaxin-1 | Eotaxin-1 | IL-12p70 | Interleukin-12p70 |
| PAI-1 (Serpin) | Plasminogen activator inhibitor-1 (Serpin E1) | Eotaxin-2 | Eotaxin-2 | IL-13 | Interleukin-13 |
| PDGF-BB | Platelet-derived growth factor | Eotaxin-3 | Eotaxin-3 | IL-15 | Interleukin-15 |
| RANTES (CCL5) | Chemokine (C-C motif) ligand 5 | FGF-2 | basic fibroblast growth factor | IL-16 | Interleukin-16 |
| Resistin (ADSF) | adipose tissue-specific secretory factor | Fractalkine | Fractalkine | IL-17A | Interleukin-17A |
| TGF alpha | Transforming growth factor alpha | G-CSF | Granulocyte colony-stimulating factor | IL-18 | Interleukin-18 |
| VCAM-1 | vascular cell adhesion molecule 1 | GM-CSF | Granulocyte-macrophage colony-stimulating factor | IL-2 | Interleukin-2 |
| VEGF-D | Vascular endothelial growth factor | Gro alpha | chemokine (C-X-C motif) ligand 1 | IL-20 | Interleukin-20 |

| Cytokine Abbreviation | Cytokine | Cytokine Abbreviation | Cytokine | Cytokine Abbreviation | Cytokine |
| --- | --- | --- | --- | --- | --- |
| IL-21 | Interleukin 21 | I-TAC | C-X-C motif chemokine 11 | SDF-1 alpha | stromal cell-derived factor 1 |
| IL-22 | Interleukin 22 | LIF | Leukemia inhibitory factor | TNF alpha | Tumor Necrosis Factor alpha |
| IL-23 | Interleukin 23 | MCP-1 | Monocyte chemoattractant protein 1 | TNF beta | Tumor necrosis factor-beta |
| IL-27 | Interleukin 27 | MCP-2 | Monocyte chemoattractant protein 2 | TNF-R2 | Tumor necrosis factor receptor 2 |
| IL-2R | Interleukin 2R | MCP-3 | Monocyte chemoattractant protein 3 | TRAIL | TNF-related apoptosis-inducing ligand |
| IL-3 | Interleukin 3 | M-CSF | macrophage colony-stimulating factor | TSLP | Thymic stromal lymphopoietin |
| IL-31 | Interleukin 31 | MDC | C-C motif chemokine 22 | TWEAK | TNF-related weak inducer of apoptosis |
| IL-4 | Interleukin 4 | MIF | Macrophage migration inhibitory factor | VEGF-A | Vascular endothelial growth factor A |
| IL-5 | Interleukin 5 | MIG | monokine induced by gamma interferon |  |  |
| IL-6 | Interleukin 6 | MIP-1 alpha | Macrophage Inflammatory Proteins- 1 alpha |  |  |
| IL-7 | Interleukin 7 | MIP-1 beta | Macrophage Inflammatory Proteins- 1 beta |  |  |
| IL-8 | Interleukin 8 | MIP-3 alpha | Macrophage Inflammatory Proteins- 3 alpha |  |  |
| IL-9 | Interleukin 9 | MMP-1 | Matrix metalloproteinase-1 |  |  |
| IP-10 | C-X-C motif chemokine ligand 10 | NGF beta | Nerve growth factor beta |  |  |

Supplementary Table 1. Cytokine abbreviations.
