# Supplementary material for: Cytokine signatures differentiate systemic sclerosis patients at high versus low risk for pulmonary arterial hypertension
Source: Arthritis Res Ther. 2022 Feb 9;24:39. doi: 10.1186/s13075-022-02734-9 (PMC8827262; doi:10.1186/s13075-022-02734-9)
Supplement: Supplementary file 4 — Additional file 4: Supplementary Table 2. Mean/standard deviation values for each cytokine by clinical group. [file 13075_2022_2734_MOESM4_ESM.docx]

| **Antigen** | **Healthy** | | **Low Risk** | | **High Risk** | | **PAH** | |
| --- | --- | --- | --- | --- | --- | --- | --- | --- |
|  | **Mean** | **Std. Dev.** | **Mean** | **Std. Dev.** | **Mean** | **Std. Dev.** | **Mean** | **Std. Dev.** |
| **IFN-beta** | 4.277 | 3.077 | 6.366 | 1.875 | 8.184 | 0.978 | 8.048 | 1.665 |
| **PAI-1** | 13.500 | 0.466 | 4.120 | 5.843 | 13.608 | 0.895 | 13.705 | 0.412 |
| **IL-1RA** | 7.180 | 2.478 | 9.731 | 0.556 | 10.296 | 0.844 | 10.181 | 1.202 |
| **Resistin** | 8.463 | 2.141 | 6.294 | 3.408 | 9.515 | 1.790 | 9.177 | 2.366 |
| **RANTES** | 0.866 | 2.192 | 5.376 | 2.530 | 7.350 | 1.802 | 7.033 | 1.925 |
| **Leptin** | 8.287 | 2.041 | 6.405 | 2.475 | 9.777 | 2.212 | 10.055 | 2.080 |
| **TGF-alpha** | 6.803 | 1.554 | 5.453 | 1.382 | 7.164 | 1.050 | 6.974 | 1.240 |
| **VEGF-D** | 8.527 | 1.207 | 8.299 | 3.062 | 10.486 | 1.717 | 10.314 | 1.886 |
| **EGF** | 9.822 | 0.699 | 6.688 | 1.644 | 9.276 | 1.472 | 9.189 | 1.471 |
| **BDNF** | 11.387 | 0.551 | 4.841 | 4.628 | 11.745 | 1.057 | 11.701 | 0.720 |
| **IL-12p40** | 1.903 | 3.453 | 7.051 | 3.235 | 9.109 | 3.162 | 9.226 | 2.770 |
| **sICAM-1** | 11.983 | 0.688 | 6.724 | 3.919 | 13.017 | 0.753 | 12.880 | 0.711 |
| **MIP1alpha** | 0.916 | 2.442 | 7.282 | 2.690 | 7.267 | 3.118 | 6.964 | 3.826 |
| **IL27** | 1.446 | 2.620 | 5.816 | 2.366 | 5.928 | 3.078 | 5.928 | 3.051 |
| **LIF** | 0.248 | 1.110 | 1.311 | 2.267 | 2.411 | 2.664 | 2.453 | 2.786 |
| **IL1beta** | 5.244 | 2.597 | 6.544 | 0.898 | 6.433 | 1.892 | 6.656 | 1.083 |
| **IL2** | 1.878 | 2.757 | 3.703 | 2.416 | 4.431 | 2.557 | 4.507 | 2.613 |
| **IL5** | 3.337 | 3.429 | 7.569 | 0.785 | 6.822 | 2.364 | 6.821 | 2.656 |
| **IL6** | 1.624 | 2.624 | 5.599 | 2.280 | 4.930 | 2.932 | 5.047 | 3.088 |
| **IL7** | 3.280 | 3.190 | 5.270 | 1.980 | 6.283 | 1.738 | 5.907 | 2.073 |
| **BLC** | 2.214 | 2.623 | 4.509 | 2.652 | 5.418 | 2.383 | 5.577 | 2.418 |
| **Eotaxin** | 3.093 | 2.980 | 6.404 | 0.822 | 6.298 | 1.982 | 6.225 | 1.925 |
| **IL12p70** | 4.209 | 2.918 | 5.603 | 2.034 | 5.995 | 2.129 | 6.070 | 2.094 |
| **IL13** | 3.532 | 3.409 | 7.059 | 0.660 | 6.825 | 1.959 | 7.183 | 1.176 |
| **IL17A** | 2.177 | 3.080 | 4.065 | 2.892 | 6.030 | 2.550 | 6.023 | 2.669 |
| **SCF** | 5.383 | 3.292 | 6.200 | 2.339 | 7.635 | 1.871 | 7.661 | 2.040 |
| **IFNg** | 3.906 | 3.402 | 5.481 | 3.124 | 6.997 | 2.076 | 6.962 | 1.800 |
| **GMCSF** | 3.231 | 2.826 | 5.499 | 2.052 | 6.263 | 1.977 | 6.214 | 1.910 |
| **TNFalpha** | 1.068 | 1.928 | 2.079 | 2.715 | 3.424 | 2.788 | 3.913 | 2.777 |
| **MIP1beta** | 0.686 | 2.212 | 6.419 | 3.491 | 7.394 | 3.437 | 7.480 | 3.655 |
| **Eotaxin3** | 2.825 | 3.234 | 5.220 | 3.709 | 7.763 | 2.611 | 7.754 | 2.613 |
| **IL9** | 2.146 | 2.778 | 6.650 | 2.548 | 7.297 | 2.759 | 7.599 | 2.153 |
| **MIP3alpha** | 2.540 | 3.220 | 6.435 | 2.366 | 7.242 | 1.979 | 7.400 | 1.807 |
| **ITAC** | 0.481 | 1.484 | 3.944 | 3.473 | 4.154 | 3.382 | 4.551 | 3.682 |
| **Fractalkine** | 1.388 | 2.547 | 7.154 | 1.614 | 6.781 | 2.940 | 6.850 | 2.743 |
| **MMP1** | 4.630 | 4.022 | 7.926 | 2.337 | 9.613 | 2.585 | 9.944 | 1.782 |
| **IL15** | 0.424 | 1.898 | 6.669 | 3.601 | 6.561 | 3.699 | 6.751 | 3.614 |
| **IL18** | 2.219 | 3.185 | 7.725 | 0.717 | 7.281 | 2.240 | 7.256 | 2.216 |
| **MCSF** | 2.577 | 3.636 | 5.433 | 3.907 | 8.769 | 2.493 | 8.976 | 2.085 |
| **MCP3** | 1.944 | 2.913 | 5.536 | 3.041 | 6.287 | 2.604 | 6.372 | 2.448 |
| **MIG** | 0.000 | 0.000 | 5.616 | 2.560 | 6.089 | 3.414 | 5.954 | 3.564 |
| **IL16** | 2.486 | 3.337 | 4.963 | 3.462 | 6.701 | 2.336 | 6.379 | 2.815 |
| **IL21** | 1.923 | 3.068 | 6.648 | 2.659 | 6.913 | 2.823 | 6.610 | 2.949 |
| **IL3** | 1.527 | 2.771 | 1.955 | 3.149 | 5.708 | 2.554 | 5.566 | 2.766 |
| **CD40L** | 2.294 | 3.517 | 8.855 | 1.196 | 7.889 | 3.246 | 7.717 | 3.680 |
| **FGF2** | 0.677 | 2.085 | 5.717 | 3.262 | 6.849 | 3.220 | 6.881 | 3.171 |
| **IL22** | 0.332 | 1.485 | 4.392 | 3.885 | 6.044 | 3.308 | 6.396 | 3.230 |
| **TSLP** | 0.678 | 2.086 | 4.979 | 2.731 | 5.091 | 2.633 | 4.947 | 3.050 |
| **IL20** | 1.718 | 2.842 | 7.119 | 1.089 | 7.495 | 2.110 | 7.298 | 2.752 |
| **ENA78** | 2.477 | 3.245 | 4.167 | 3.299 | 7.111 | 2.344 | 6.978 | 2.696 |
| **CD30** | 4.519 | 3.169 | 5.989 | 1.785 | 6.631 | 1.325 | 6.603 | 1.483 |
| **TNFRII** | 5.913 | 2.892 | 7.444 | 1.408 | 9.183 | 2.148 | 9.434 | 0.964 |
| **BAFF** | 1.763 | 2.511 | 6.103 | 2.206 | 5.096 | 2.872 | 5.504 | 2.802 |
| **MDC** | 1.235 | 2.413 | 7.096 | 0.705 | 6.544 | 2.662 | 6.251 | 2.830 |
| **APRIL** | 1.938 | 3.127 | 5.154 | 2.918 | 6.579 | 2.493 | 7.051 | 1.952 |
|  |  |  |  |  |  |  |  |  |
|  |  |  |  |  |  |  |  |  |

Supplementary Table 2. Mean/standard deviation values for each cytokine by clinical group.
